# Pre-service Chemistry Teachers’ Views about the Tentative and Durable Nature of Scientific Knowledge

**DOI:** 10.1007/s11191-022-00374-8

**Published:** 2022-08-27

**Authors:** Stefan Mueller, Christiane S. Reiners

**Affiliations:** Institute of Chemistry Education, Faculty of Mathematics and Natural Sciences, University of Cologne, Cologne, Germany

## Abstract

With regard to current controversial public discussions about the credibility of scientific knowledge, it seems particularly important that students possess adequate ideas about the tentativeness of scientific knowledge, which is a key aspect of nature of science. However, international studies show that many pre-service science teachers tend to have naïve conceptions about the tentativeness and these conceptions turn out to be resistant to change. So far, no research was done, on the conceptions of German pre-service chemistry teachers about tentativeness. Therefore, two empirical, qualitative research studies were conducted. The first study with 50 participants was to investigate, which conceptions about tentativeness German pre-service chemistry teachers possess, what the origins of these conceptions are and if they are resistant to change. In a second study with 56 participants, it was examined how a more adequate and functional understanding could be promoted. Data were collected by using different methods, such as open-ended questionnaires and semi-structured interviews. The participants’ views about tentativeness were assigned to different categories. Results show that most participants held inconsistent or only partially informed views on tentativeness. The views turn out to be resistant to change, and many participants are not able to explain their ideas. And if so, their explanations are mostly restricted to scientific theories. Additionally, dealing with tentativeness unsettles some participants. To promote an adequate understanding, new approaches were developed, like the BlackTube activity. Additionally, instructions should focus on the durability of scientific knowledge. Furthermore, a differentiated reflection on different types of scientific knowledge seems necessary.

## Introduction

With regard to current controversial public debates about the credibility of science (“fake science”, “alternative facts”), dealing with the reliability but also with the tentativeness of scientific findings is a crucial task of science education. Therefore, Kampourakis (2018) states: “It seems that the main problem is not only the lack of understanding of the relevant science, but also—and perhaps most crucially—the lack of understanding of the uncertainty inherent in science” (p. 830). The importance of such an “understanding of uncertainty” was recently demonstrated in discussions about the frequently changing findings on COVID-19, especially at the beginning of the crisis. Accordingly, the COVID-19 pandemic is now considered a socioscientific issue (Maia et al., 2021; Moura et al., 2021; Reiss, 2020), “whose discussion of social, political, economic, and ethical aspects may support students’ learning of nature of science, thus fostering scientific literacy” (Maia et al., 2021, p. 1075). Additionally, an adequate understanding of the tentative and durable nature of science is also important with regard to other socioscientific issues such as climate change or the expansion of renewable energies. In the latter two examples, content knowledge about chemistry is for the upmost importance.

To address these challenges for science and especially for chemistry education, the concept of tentativeness is first examined from various theoretical perspectives (see Section 2). This approach culminates in a guiding framework of the “tentative and durable nature of science” for science teacher education (see Section 2.4). The presentation of the current state of research (see Section 3) leads to research questions that are answered with the help of two qualitative studies. The main results of these investigations are presented in Sections 4 and 5 and discussed in Section 6.

## Tentativeness of Scientific Knowledge

In this chapter, tentativeness of scientific knowledge will be regarded first from selected philosophical positions (see Section 2.1) and then from the perspective of science education (see Section 2.2). Based on these different viewpoints, a guiding framework of the tentativeness and durability of scientific knowledge for teacher education is presented in Section 2.4. This definition should help to address the current discussions about tentativeness and durability of scientific knowledge within science education and forms a foundation for the empirical investigations.

### Tentativeness—from the Perspective of Philosophy of Science

From the viewpoint of philosophy of science,^1^ the general tentativeness of all scientific knowledge can be justified with Karl Popper’s (1959) principle of falsification. According to Popper, an essential characteristic of scientific research is to establish and test propositions or systems of propositions. He states that hypotheses and systems of theories would be developed and tested against experience through observations and experiments. A scientific claim that is not yet refuted by (empirical) investigations is temporarily approved. However, the theory could never be called finally true or absolutely verified, because it is never possible to empirically test all conceivable individual cases (Popper, 1963). Thus, every scientific theory and theorem is tentative, since a certain residual uncertainty always remains. Popper (1959) illustrates this with the help of a famous analogy about the observations of swans: “No matter how many instances of white swans we may have observed, this does not justify the conclusion that all swans are white” (p. 27). While a final verification is not possible by any number of experiments, scientific findings could be disproved at any time by a new, decisive experiment, a so-called “experimentum crucis” (ibid.). Such an experiment, he argues, is particularly suitable for bringing a decision between two competing theories by disproving (at least) one of them without thereby proving the other. Popper sees this falsifiability as a criterion to confirm the scientific nature of a claim and of demarcation from non-scientific statements. After all, it should be possible to refute any scientific knowledge based on experience.

It should be noted that the tentative nature of scientific knowledge could also be considered and explained from many other perspectives and theories from philosophy of science (e.g. the “Structure of Scientific Revolutions” by Thomas Kuhn (1962), the “Anarchistic Theory of Knowledge” by Feyerabend (1975), the “strong programme in the sociology of scientific knowledge” (Bloor, 1976), Hacking’s “experimental realism” (Hacking, 1983) or the Bayesian epistemology (Olsson, 2018)). In this case, however, the plurality of representations is dispensed in favour of the basic principle of falsification, which is often referred to in science education research (cf. Section 2.2). Nevertheless, some points of criticism and comments are described briefly below.

Despite its wide distribution, some aspects of Popper’s principle of falsification are discussed controversial within philosophy of science. One counterargument is that Popper would describe an ideal of science that cannot be achieved in reality. For example, Imre Lakatos (1976) criticizes Popper’s, in his opinion, “naïve falsificationism” (p. 93). Lakatos doubts the existence of an “experimentum crucis”, since scientific knowledge would not be abandoned immediately by a single experiment (ibid.). Additionally, he states that the description of experiments as “decisive” can only be determined retrospective; thereby, he gives falsification a “historical character”. Moreover, he believes that scientific theories could not be falsified based on a single observation or experiment, because scientific knowledge is embedded into a broad network of interdependent findings, theories and assumptions (ibid.). Thus, in the case of a contradiction between theory and observational data, it is not possible to decide which assumptions are false and must be discarded accordingly (Chalmers, 1976). This logical problem of falsification is also found in the “Duhem-Quine thesis” (Chalmers, 1976; Singham, 2020), according to which theories are underdetermined by observational data. Furthermore, Lakatos (1976) emphasizes the role of the scientific community for the recognition and falsification of findings. While Lakatos takes a very rationalistic view in this respect, Kuhn (1962), as a representative of modern relativism (Kircher, 2015), emphasizes also the crucial importance of sociological and psychological factors on “paradigm shifts” (Kuhn, 1962). According to Kuhn, a “scientific revolution” occurs, whenever a new paradigm displaces a prevailing paradigm. As the term “revolution” indicates, supporters of two rival paradigms compete with each other, whereby not only logical arguments and empirical data are used, but also social and sociocultural factors play a key role. Another difference between Popper’s and Kuhn’s descriptions of scientific transformations is that Popper (especially in his early publications) sees change of scientific knowledge as progress, whereas Kuhn takes the view that paradigm shifts do not lead to getting closer to truth (Maeder, 2020). Instead, statements of different paradigms could not be compared at all, as they represent different approaches to explain the world (ibid.). Two paradigms are thus “incommensurable” (Kircher, 2015, p. 826).

Bell (2006) summarizes the aforementioned considerations by regarding different characteristics of scientific knowledge as explanations for their tentativeness: In line with Popper’s principle of falsification, scientific findings could be rejected on the basis of new empirical evidence (Bell, 2006). These new findings could be based on technological progress, such as new and more accurate measuring devices, or on conceptual progress, for example due to the development of new theoretical ideas to set up an experiment. Accordingly, the empirical nature of science could be considered as a reason for the tentativeness of scientific knowledge. On the other hand, scientific knowledge could also be revised due to a reinterpretation of existing evidence in light of new ways of thinking (ibid.). In line with Kuhn’s previously described ideas about paradigm shifts, Bell (2006) states that such reinterpretations could also happen because of changes in the sociocultural sphere. Thus, the sociocultural embeddedness of scientific knowledge as well as the subjectivity of researchers could also be seen as reasons for the tentative nature of science (ibid.). Furthermore, the role of creativity and human imagination could play a major role when scientific knowledge changes, because scientific knowledge is not a product of only logical and rational considerations of scientists. Instead, innovative methods and inventions of creative explanations are necessary for their acquisition, but also for their refutation (ibid.). The distinction between observation and conclusion could also be regarded as origin of tentativeness, since both ultimately lead to different types of scientific knowledge, namely theories and laws, which are both principally tentative (ibid.; McComas, 1998; Reiners et al., 2017). Bell (2009) concludes that all of the previously described aspects of nature of science (NOS) are interrelated. Furthermore, the other aspects of NOS could be regarded as reasons for tentativeness, which he considers to be the central characteristic of scientific knowledge: “However, closer consideration reveals that they [the other aspects of NOS; author’s note] all fall under the umbrella of tentativeness: There are no ideas in science so cherished or privileged as to be outside the possibility of revision, or even rejection, in light of new evidence and new ways of thinking about existing evidence” (Bell, 2009, p. 3).

In summary, the principle of falsification shows that tentativeness is a central characteristic of scientific knowledge. Moreover, if falsification is considered a crucial criterion of demarcation between science and pseudoscience (Pigliucci & Boudry, 2013), it becomes clear that an adequate understanding of tentativeness is also very important in current discussions about “fake science” in public. Accordingly, the aspect of tentativeness is of increasing importance in the context of science education (research).

### Tentativeness from the Perspective of Science Education Research

Although there is no consensus in science education research about which aspects belong to “nature of science” (NOS), tentativeness is, among others, named as one important feature of scientific knowledge in most definitions and frameworks (Neumann & Kremer, 2013; cf. Müller & Reiners, 2020b). For example, Lederman et al., (2002, p. 499 ff.) identify the following seven characteristics of scientific knowledge:

- The empirical nature of scientific knowledge and the distinction between observations and inferences
- The functions of, and relationship between, scientific theories and laws
- The creative and imaginative nature of scientific knowledge
- The theory-laden nature of scientific knowledge
- The social and cultural embeddedness of scientific knowledge
- The myth of “the scientific method”
- The tentative nature of scientific knowledge

Furthermore, tentativeness is often highlighted as a particularly central aspect of nature of science (Bell, 2009). Accordingly, it has been identified as a component of numerous science education standard documents and curricula in various countries, as shown by McComas and Olson (1998) and Olson (2018), as well as Summers et al. (2019). Additionally, scientists themselves frequently describe it as a characteristic of scientific knowledge (Wong & Hodson, 2009).

Moreover, tentativeness was also highlighted as a central aspect of scientific knowledge in the widely regarded Delphi study by Osborne et al. (2003). In the study, the interviewed experts conclude that learners should be taught that although scientific knowledge is often confirmed, it remains always subject to change in the future due to new findings or due to the reinterpretation of previous findings. This description of tentativeness shares many similarities to numerous other definitions from international NOS literature, which are used to evaluate textbooks (Abd-El-Khalick et al., 2008; Marniok & Reiners, 2017; Vesterinen et al., 2013) or to assess learners’ and teachers’ views about NOS (Chen, 2006; Halloun & Hestenes, 1998; Lederman et al., 2002; Liang et al., 2009). Most of these descriptions emphasize that scientific knowledge can change because of both new findings and reinterpretation of existing knowledge (Abd-El-Khalick et al., 2008; Chen, 2006; Lederman et al., 2002). In addition to the creative, interpretative and sociocultural nature of science, tentativeness is also justified by the principle of falsification (see also Bell, 2006; Lederman, 2006). Although all scientific findings were described as principally tentative in many descriptions, theories and laws as well as “facts” are usually mentioned explicitly (Abd-El-Khalick et al., 2008; Lederman et al., 2002). With regard to the tentative nature of chemical knowledge, Tolvanen et al. (2014) point out that it “includes also change in chemical instruments and creation of new substances” (p. 1614). Referring to the work of Popper and Kuhn (cf. Section 2.1), Chen (2006) emphasizes that changes in science can be both evolutionary and revolutionary (see also McComas & Olson, 1998). Likewise, Höttecke and Hopf (2018) distinguish between slow, i.e. gradual, changes and scientific revolutions.

However, descriptions of tentativeness, like the one from Lederman et al. (2002), as components of the so-called consensus-view (Allchin, 2013), are also criticized by representatives of other modelling approaches of NOS. For example, it is criticized that the changeability of scientific knowledge is often over-emphasized in the form of tenets and catchwords like “tentativeness” or “subject-to-change” in consensus lists of NOS. However, such a description is considered a factually inappropriate representation of such a complex aspect (Clough, 2007). Moreover, such an abbreviation could unsettle learners. In worst case, addressing the tentative nature of science could even inadvertently lead students to mistrust scientific knowledge (Cobern, 2020; Cobern et al., 2022; Hodson & Wong, 2017). In this regard, Romero-Maltrana et al. (2019) are warning that a possible misinterpretation of the common view of NOS tenets could lead to epistemic relativism.

In order to avoid confusions among learners, Allchin (2013) recommends teaching tentativeness not only by a NOS tenet-list, but also within a framework of holistic approaches and connected to other NOS aspects. Accordingly, Allchin (2011) points out that the isolated fact that scientific knowledge is tentative would not help learners to make everyday decisions. In his view, it is more important to enable learners to evaluate the reliability and credibility of scientific statements in their daily life (ibid.). Instead of presenting a list of tenets, this should be achieved by dealing with historical and contemporary examples from science, which depict nature of science and scientific inquiry as a whole (ibid.). On the other hand, Cobern (2020) suggests that while teaching the tentativeness of scientific knowledge, equal emphasis should be placed on teaching its durability, credibility and certainty in order to prevent learners from mistrusting scientific knowledge and to avoid confusions.

### Durability of Scientific Knowledge

The reliability and durability of scientific knowledge are both mentioned in the definitions of tentativeness by Abd-El-Khalick et al. (2008), by Chen (2006) and by Lederman et al. (2002). However, in contrast to the respective description of tentativeness, they are not further elaborated. Bell (2009) explains that it is reasonable to trust scientific findings despite, or precisely because of, their principle tentativeness, because they have been tested empirically many times and have nevertheless been valid for a long time: “Scientific knowledge, once generally accepted, can be robust and durable. Many ideas in science have survived repeated challenges, and have remained largely unchanged for hundreds of years. Thus, it is reasonable to have confidence in scientific knowledge, even while realizing that such knowledge may change in the future.” (p. 3). Bell (2009) believes that tentativeness should not be seen or communicated as a weakness of science; instead, he regards it actually as one of its greatest strengths and as a characteristic feature of science that allows the continuous progress of science “towards legitimate claims and away from erroneous” (p. 4). Finally, the advances of science, which are based on constant reviews, and the application of scientific findings would ultimately show that scientific knowledge is the most successful and credible knowledge of humankind. Likewise, McComas (2020) concludes that “scientific knowledge is tentative and self-correcting but ultimately durable” (p. 59).

Kampourakis (2018) also emphasizes that the tentative nature of science on the one hand would not allow any conclusions about the durability of scientific knowledge on the other hand. Furthermore, the principle tentativeness of scientific knowledge could also be regarded as a motivation for further research: “This, of course, does not mean that science in general is uncertain. On the contrary, most of our scientific understanding is solid and robust as far as the general picture is concerned. There is no doubt that evolution and climate change are happening, or that vaccines are overall useful and safe. However, there will always be uncertainties in the details. These uncertainties motivate further research and […] uncertainty actually makes science advance.” (Kampourakis, 2018, p. 830). Singham (2020) sees a “Great Paradox of Science” (p. 269) in the fact that, although scientific knowledge cannot be proven absolutely and therefore do not represent definitive truth, it is very successful in explaining the world and in making new technologies possible. As reasons for the reliability and workability of scientific knowledge, Singham names empirical evidence and its systematic evaluation, creation of consensus conclusions within the scientific community as well as peer-reviewed publications (ibid.). As a further reason for the consistency and reliability of scientific knowledge, Höttecke and Hopf (2018) argue that theories and models could retain their explanatory power in certain fields of science, even if they already have been extended or replaced by other models.

Following the above considerations, learners should also be taught about the durability, reliability and credibility of scientific knowledge in line with its tentativeness in order to evaluate and classify scientific statements in everyday life. Therefore, Cobern (2020) calls for the development of new approaches for teaching the tentativeness of science, which include the durability of knowledge, as well as new approaches for teaching the durability that also include tentativeness. As a condition for these approaches, he demands a much better understanding of how students interpret and apply the idea that scientific knowledge is tentative vis-à-vis the durability of scientific knowledge (ibid.). To avoid that students mistrust science, because of its tentativeness, Cobern et al. (2022) suggest science educators to increase “their instructional focus on the relationship between data and evidence that leads to the durability of scientific knowledge, and on how that exists in balance with the concept of tentativeness” (p. 26). Another approach is presented by Clough (2007), who suggests that students should explore NOS features in a form of questions, rather than through presenting the features as a list. Accordingly, students’ understanding of tentativeness should be promoted by asking them questions about the extent to which scientific knowledge is tentative and the extent to which it is durable. In this way, the durability of scientific knowledge would be placed on an equal footing with its tentativeness.

Based on the presented viewpoints from philosophy of science and science education research, a guiding framework of the tentativeness and durability of scientific knowledge for science teacher education was developed, which is presented and discussed in the following section.

### Guiding Framework of the Tentative and Durable Nature of Science

**Fig. 1:**
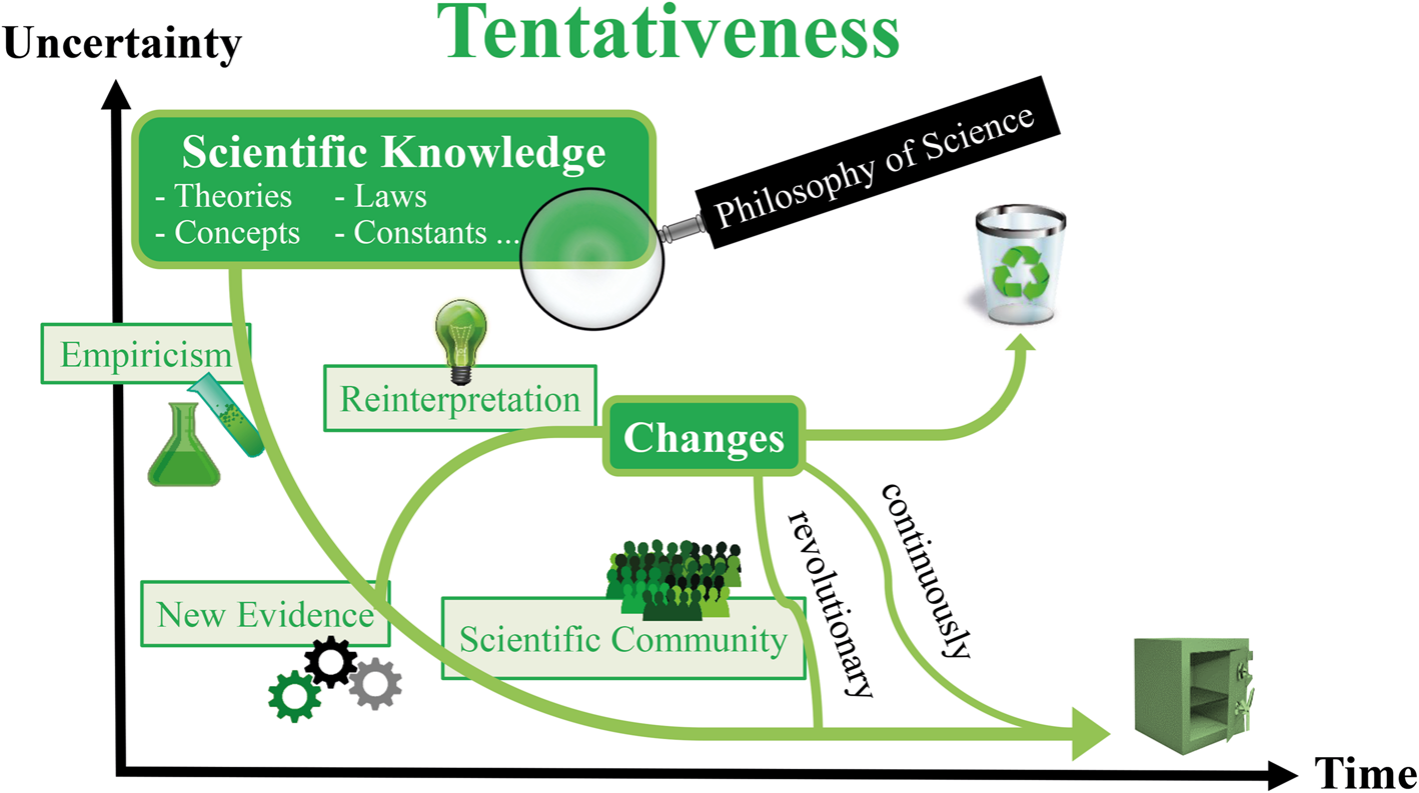
A scheme visualizing the tentative and durable nature of scientific knowledge (see Müller & Reiners, 2020b)

The following guiding framework of tentativeness and durability of scientific knowledge (see also Müller, 2021; Müller & Reiners, 2020b), which is also visualized in a diagram shown in Fig. 1, is fundamental for the conducted studies:

Scientific knowledge becomes reliable more and more over time, because it is empirically tested,^2^ valid for a long time and therefore very robust to changes (Osborne et al., 2003). Accordingly, scientific knowledge is widely accepted within the scientific community. In addition, most scientific theories have a high explanatory value and are almost free of inconsistencies. In this regard, scientific knowledge is durable (Abd-El-Khalick et al., 2008). However, all categories of scientific knowledge (theories, laws, constants, etc.) cannot be proven absolutely. Therefore, they are tentative as they are principally all subject to change (Popper, 1959). These changes can take place slowly and continuously or revolutionary (McComas & Olson, 1998). Scientific knowledge can change when new evidence and knowledge arise by conceptual or technological progress. Changes might also be due to reinterpretation of existing evidence and knowledge in light of new theoretical ideas or to changes in the cultural and social spheres (Lederman et al., 2002). It depends on the scientific community whether scientific knowledge is rejected, changed or partly changed (Kuhn, 1962). The principal tentativeness of scientific knowledge thus does not imply arbitrariness or unreliability of science. Far from it, the ongoing critical review and discussions by scientists, as well as the rejection of obsolete findings, underline the durability and reliability of accepted knowledge. In this way, scientists are able to gain reliable knowledge approximately (Bell, 2009).

The above framework is primarily addressed to in-service teachers and pre-service teachers. Accordingly, it is based on the so-called consensus definitions of tentativeness (Lederman et al., 2002; Osborne et al., 2003; cf. Section 2.2). However, it also takes the additions and criticisms into account, which are described in Section 2.1. For example, the durability of scientific knowledge is emphasized and elaborated in the framework itself and in its title. This should ensure that learners do not mistrust scientific findings (Cobern et al., 2022) and consider science neither completely unchangeable nor untrustworthy (cf. Sections 2.2 and 2.3). The guiding framework also subsumes other aspects discussed above, such as the great influence of the scientific community on the recognition of findings or the diversity of reasons for changes of scientific knowledge. The process of gaining scientific knowledge and different types of scientific knowledge should also be taken into account. In order to avoid any unsettlement of learners, the often negatively connoted term “tentativeness” (Lederman, 2006) is rarely used within the framework and is contrasted with durability in the sense of Clough (2007, cf. Section 2.3) ideas.

Moreover, following Allchin’s (2011) comments, the guiding framework is not intended to be passed on to students as declarative knowledge. Instead, it is rather a proposal and support for teachers to design competence-oriented learning environments. In this way, learners should be able to participate actively in discussions about the certainty and trustworthiness of scientific knowledge. This will be illustrated by the following example of discussions about the causes of climate change: In order to assess, whether publications and studies on climate change meet scientific standards, students can refer to the criterion of falsifiability. Although the causes of climate change are controversially discussed in public media and although they can never be proven absolutely, there has been consensus of the courses within the scientific community for a long time (Cook et al., 2013; Oreskes, 2004). Therefore, students should recognize that this consensus about anthropogenic influences on climate change is based on comprehensive, empirical research. In this context, the principle tentativeness of scientific research can also be regarded as an opportunity for the development of sustainable innovations (Müller & Reiners, 2021).

The approximation of scientific knowledge to a certain status in the course of time and through continuous empirical validation is described at the end of the guiding framework. It is also illustrated in the associated diagram (see Fig. 1): The arrow, which represents the development of scientific knowledge, approximates the abscissa of the depicted coordinate system, but never touches it, because a residual uncertainty always remains. This is further symbolized by a picture of a vault at the end of the arrow. Thus, the illustrated vault is wide open to show that it offers only an illusion of absolute security. In addition, it should be noted that the time axis in Fig. 1 is in no way meant to imply that the uncertainty of scientific knowledge automatically decreases with time. Instead, scientific knowledge may become increasingly durable over time through empirical validation or due to the (re-)interpretation and discussion by the scientific community. Another symbol used in the diagram is that of a recycling bin. It is intended to show that even scientific knowledge, which was initially rejected by the scientific community, could be “recycled” and reappear again in a modified form.

## Pre-service Teachers’ Conceptions About Tentativeness

Numerous instruments have been developed and used to identify and assess students’, pre-service teachers’ and in-service teachers’ ideas about nature of science. Most of them include questions about the tentativeness of scientific knowledge. This chapter summarizes the state of research on pre-service teachers’ conceptions about tentativeness of scientific knowledge (see Section 3.1) as well as research on possible origins of these conceptions (see Section 3.2). Based on these descriptions, research questions are derived (see Section 3.3).

### State of Research on Pre-service Teachers’ Conceptions About Tentativeness

In his article “The Principal Elements of the Nature of Science: Dispelling the Myths” (McComas, 1998), William F. McComas lists 15 myths about nature of science, which, from his point of view, are widespread among students, but also among adults (ibid.). Among these myths are several naïve ideas about the tentative nature of scientific knowledge. For example, students tend to believe that scientific laws and other ideas like that were absolute; that carefully accumulated evidence would result in sure knowledge; and that science and its methods would provide absolute proof (ibid.). Moreover, some other myths from McComas’ list are related to tentativeness. For instance, students might think that scientific models represent reality and that a hierarchical relationship exists between scientific hypotheses, theories and laws (ibid.). According to this last and common myth, scientific research would start with a hypothesis, which would become a theory with gathering evidence and would turn into a scientific law upon absolute “proof” (Horner & Rubba, 1979; Maeng & Bell, 2013; Reiners et al., 2017). From an epistemological perspective, in contrast, laws are not “mature” theories as they represent different types of scientific knowledge, which serve completely different functions (Reiners et al., 2017). While scientific laws can be described as generalizations and descriptions of observations and observational data, theories offer explanations and predictions of phenomena (ibid.). In a study with 153 participants, Abd-El-Khalick (2006) empirically confirms that many college students consider theories to be tentative (77% of responds), while laws are mostly regarded as absolute and unchangeable (90% of responds). Additionally, students describe theories as preliminary stages of laws that have not been “proven” yet (ibid.). The aforementioned myths suggest that an investigation of students’ conceptions of tentativeness has to differentiate precisely between various types of scientific knowledge, as some types will be more likely considered to be tentative than others.

In their meta-study, Cofré et al. (2019) analysed articles from 2000 to 2018 in which conceptions about nature of science were collected before and after an intervention. In total, they regarded 52 articles from nine different scientific journals.^3^ Overall, the authors conclude that some aspects of NOS appear to be easier to learn and to understand than other aspects (ibid.). For example, many participants in several studies already held informed views about both the empirical and the creative nature of science before the respective intervention (ibid.). Moreover, both aspects, and in addition the distinction between observation and inference, were relatively easy to learn through appropriate instructions. In contrast, the tentative nature of science and its sociocultural embeddedness, as well as the characteristics of scientific theories, laws and models, seem to be much more difficult for most learners to understand (ibid.). However, it should be noted that the regarded studies are different in many ways: conceptions from diverse groups of people (students, pre-service teachers and in-service teachers) were investigated with regard to various aspects of nature of science and with different methods (ibid.). With regard to pre-service science teachers’ conceptions, a total of 16 journal articles were considered, nine of which related to studies with pre-service primary teachers and seven to those with pre-service secondary teachers. Between five (McDonald, 2010) and 75 (Bell et al., 2011) pre-service teachers participated in each of these studies (the average number of participants is nearly 28). While conceptions about tentativeness were collected in 15 of the 16 studies (Cofré et al., 2019), tentativeness represented a NOS aspect with the most “naïve” pre-test results in four studies (Abd-El-Khalick & Akerson, 2004, 2009; Lin & Chen, 2002; Matkins & Bell, 2007). Moreover, two studies conclude that pre-test beliefs about tentativeness are among the least likely to change ideas about NOS (McDonald, 2010; Ozgelen et al., 2013). However, in three studies, most pre-service teachers also held informed views of tentativeness in the pre-test (Akerson et al., 2006; McDonald, 2010; Schwartz et al., 2004). Additionally, there was a comparatively large improvement of participants’ conceptions regarding this aspect of NOS during two of the regarded studies (Abd-El-Khalick & Akerson, 2004; Akerson et al., 2000). Thereby, explicit and reflective interventions turn out to be most helpful in promoting understanding about tentativeness (Abd-El-Khalick & Akerson, 2004; Akerson et al., 2000) and about nature of science in general (Abd-El-Khalick & Lederman, 2000; Bell et al., 2011; Khishfe & Abd-El-Khalick, 2002; Yacoubian & BouJaoude, 2010).

Due to the diversity of the studies considered, no clear picture about conceptions emerges from the meta-study. Nevertheless, it shows in summary that many pre-service science teachers only possess naïve views about the tentativeness of scientific knowledge. Mesci and Schwartz (2017) investigated in an explorative study, which conceptions of pre-service science teachers about nature of science are more or less likely to change. In their study with pre-service science teachers (including six participants studying to become primary school teachers), five of the 14 participants hold naïve views about tentativeness at the beginning (ibid.). For example, one participant was of the opinion that scientific laws could not change anymore: “I think scientific laws remain laws because they have been proven scientifically correct by evidence and they will remain laws forever. (Aaron, Interview-Pre)” (ibid., p. 339). In comparison to the pre-test, only one-half of participants were able to attain views of tentativeness at a higher level between “naïve” and “informed + + +” due to the intervention. Three pre-service teachers were even assigned to a lower level (ibid.). Therefore, Mesci and Schwartz conclude that pre-service science teachers’ conceptions about the tentativeness of scientific knowledge (along with conceptions about its sociocultural embeddedness and the distinction between laws and theories) are more resistant to change than conceptions about other aspects of NOS. The latter include the subjective, the creative and the empirical nature of science as well as the distinction between observation and inference (ibid.). Mesci and Schwartz (2017) identified several factors, which might be related to pre-service science teachers’ views of tentativeness and why these views are less likely to change. Among them are instructional factors. For instance, pre-service teachers who struggled with the tentative nature of science expressed a need for more examples from both historical and contemporary science and requested more hands-on activities, as well as classroom discussions, and readings (ibid.). Additionally, Mesci and Schwartz (2017) point out the importance of sociocultural and motivational factors for conceptual change. For example, those pre-service teachers, who saw a relevance of tentativeness of scientific knowledge in terms of their future teaching, experienced a greater increase. Likewise, Abd-El-Khalick and Akerson (2004) name motivational factors as crucial when it comes to conceptual change about tentativeness and NOS in general (ibid.). Nevertheless, due to different results and conditions of existing studies, further research is necessary to clarify, which methods, instructions and examples are most appropriate to promote adequate understanding about tentativeness.

The studies presented so far are mostly qualitative studies with relatively small samples (11 of the 16 studies considered were conducted with fewer than 30 participants). In an international comparative study on pre-service teachers’ conceptions, Liang et al. (2009) analysed a significantly greater sample. In their study with 640 pre-service science teachers from three different countries, Liang et al. find out that the majority of participants possess relatively informed views about the tentativeness of certain scientific knowledge. However, results reveal significant differences between pre-service teachers from China (which are particularly informed about tentativeness) and those from the USA and Turkey (ibid.). However, it should be noted that all items of the used SUSSI questionnaire (“Students Understanding of Science and Scientific Inquiry”) only refer to the tentative nature of scientific theories, while the changeability of other types of knowledge is not included, such as the tentativeness of scientific laws. Therefore, the study is not appropriate to assess pre-service teachers’ views about tentativeness in its entirety. Moreover, participants rarely gave any answers to the open-ended questions. They mainly justified the tentativeness of scientific theories on the basis of finding new evidence using new technology. The reinterpretation of existing evidence was rarely named as an argument for tentativeness (ibid.).

Cobern et al. (2022) investigated views of about 500 pre-service, elementary/middle school teachers on the tentativeness and trustworthiness of science. For this purpose, the participants were asked to respond on noncontroversial and controversial statements about science. The former were drawn from physics and biology. Cobern et al. (2022) found that almost all participants embraced the tentative nature of science. However, regarding the trustworthiness of science, many participants “were not willing to say that they trust scientific knowledge” (Cobern et al., 2022, p. 1). As an explanation for this mistrust in science, many of them repeated that scientific knowledge is tentative.

Bektas et al. (2013) investigated pre-service chemistry teachers’ conceptions about the tentativeness of scientific knowledge at a Turkish university. In the study, most participants hold “transitional” views (5 out of 7 participants) prior to the intervention, since their views could not be classified as either “naïve” or “informed”. This could be explained by the fact that most pre-service teachers agreed with the tentativeness of science but did not explain their views (ibid.). In contrast, after the intervention, five of seven participants exhibited informed views of tentativeness and no participant possessed naïve ideas anymore. As an example of tentative scientific knowledge, the participants almost exclusively referred to the development of atomic models (ibid). For chemistry teacher education, Bektas et al. (2013) recommend to create awareness of (mis)conceptions about NOS and of the origins of such conceptions (ibid.).

With regard to German-speaking countries, there are no specific studies focussing on pre-service chemistry teachers’ conceptions about tentativeness so far. However, in their study, Höttecke and Riess (2007) interviewed 10 pre-service physics teachers. They discovered that although participants held partially adequate views at the beginning of their study, these views are often inconsistent. For example, the idea that scientific knowledge can be proven absolutely could be paired with the opinion that scientific knowledge is principally tentative (ibid.). Furthermore, the principle of falsification is hardly known among pre-service teachers, whereas the idea of a hierarchical relationship between theories and laws as well as the opinion of “unchangeable” scientific laws seems to be very common. A study by Reinisch and Krueger (2018) about pre-service biology teachers’ conceptions about the tentative nature of scientific theories shows that the ten participants surveyed can provide arguments for both the tentativeness and the durability of theories and models. Conceptions about the changeability of other types of knowledge were not considered in this case.

### Origins of Conceptions About Tentativeness

McComas (1998) suggests textbooks as well as a lack of philosophy of science content in teacher education to be the origins of the common myths about science. Abd-El-Khalick et al. (2008) also name textbooks, teacher education at universities and teacher traineeships as central sources of naïve ideas about nature of science. The lack of representations of the tentative nature of science in textbooks in German-speaking countries is confirmed by a study from Marniok and Reiners (2017). In addition, explanations of nature of science within the textbooks examined are mostly limited to explanations of models and their characteristics (ibid.).

In the study by Bektas et al. (2013), the participants predominantly named their former science teachers, textbooks and their own experiences as origins of their ideas about tentativeness of scientific knowledge. Students’ ideas about science in general can also be traced back to influences of daily media, such as advertisement, movies, cartoons and literature for children (Höttecke & Hopf, 2018; Sahin & Koeksal, 2010; Song & Kim, 1999). However, little is known about how movies and entertainment television actually influence peoples’ opinions and attitudes towards science (Weingart, 2017). Furthermore, an influence of news broadcasts on conceptions about sociocultural embeddedness of scientific knowledge has been demonstrated among non-science majors (Leung et al., 2017). It is questionable whether these daily media also exert a direct influence on the image of pre-service chemistry teachers about tentativeness of scientific knowledge.

### Research Questions and Overview of Research Studies

In the studies presented in the Sections 3.1 and 3.2, different aims are pursued and different methods are used. Therefore, results about pre-service science teachers’ conceptions about tentativeness of scientific knowledge are ambiguous. In summary, however, it can be concluded that before an intervention, some pre-service science teachers possess naïve or inconsistent views about tentativeness of scientific knowledge. According to the study by Liang et al. (2009), the majority of pre-service teachers seem to accept scientific theories as tentative. However, this does not indicate that the majority of pre-service science teachers have informed views about the tentative nature of scientific theories. For example, most participants of the study by Liang et al. (2009) held the naïve conception that a scientific theory could be proven to became a scientific law. Therefore, it is reasonable to assume that pre-service science teachers’ naïve or inconsistent views about tentativeness refer rather to other types of knowledge, such as scientific laws, which learners tend to regard as absolute and unchangeable (Abd-El-Khalick, 2006; McComas, 1998). Moreover, pre-service teachers are often not able to justify or explain the tentative nature of science (Bektas et al., 2013; Höttecke & Riess, 2007). Mostly, they explain it in reference to new findings and less often in terms of the reinterpretation of existing knowledge (Akerson et al., 2000; Bektas et al., 2013). Although there are different results in this regard, pre-service teachers’ views could be promoted with the help of an explicit and reflective intervention about NOS. Nevertheless, the investigation from Mesci and Schwartz (2017) indicates that pre-service science teachers’ conceptions of tentativeness are especially resistant to change compared to conceptions about other aspects of NOS. While most of the presented studies analysed conceptions of in-service and pre-service science teachers in general, there are relatively few studies dealing specifically with views of pre-service chemistry teachers about the tentative nature of science. Such an investigation seems necessary, because chemical knowledge plays a crucial role to understand contemporary challenges such as climate change or the expansion of regenerative energy sources. Therefore, adequate ideas of the strengths and limitations of this knowledge are essential for students to participate in public debates. Furthermore, due to the results by Liang et al., it seems necessary to take into account the cultural context. Thus, it still needs to be clarified to what extend pre-service chemistry teachers in Germany also tend to have naïve or inconsistent views about tentativeness and whether these conceptions are resistant to change. Additionally, it is desirable to investigate the origins of the conceptions as well as the possible reasons for resistance to change in order to design suitable learning environments to promote pre-service chemistry teachers’ understanding. Based on these considerations, the following research questions arise:

1. Which conceptions about the tentativeness of scientific knowledge do German pre-service chemistry teachers possess?
2. What are the origins of these conceptions?
3. Are these conceptions resistant to change?
4. How far are school-related contexts and NOS activities suited to promote a more adequate and more functional understanding of tentativeness among future chemistry teachers?

To answer the research questions, two qualitative studies were conducted. While the first study was designed to answer the first three research questions, study 2 should serve primarily to answer the fourth question. An overview of the participants, design and data collection methods of both studies is shown in Table 1. Further information and main results of the studies are presented in the following chapters.

**Table 1:** Overview of participants, data sources and topics of the intervention units of both research studies

Participants	Data sources	Topics of the intervention units
Study 1 (research questions 1, 2 and 3 were investigated)		
• 50 participants • 24 female, 24 male, two did not specify • Average age: 22.9 years	Pre-test: • Questionnaires • Semi-structured interviews During the intervention (5 weeks): • Participatory observation • Portfolios • Classroom artefacts Post-test: • Questionnaires • Semi-structured interviews Follow-up test: • Semi-structured interviews (6 to 12 months after the intervention)	1) A general introduction to nature of science 2) Case studies from the history of science to illustrate the sociocultural embeddedness of scientific knowledge 3) Reflection on the tentativeness of scientific knowledge 4) Non-contextualized activities 5) Application to chemistry teaching
Study 2 (research question 4 was investigated)		
• 56 participants • 29 female, 26 male, one person did not specify • Average age: 21.5 years	Pre-test: • Questionnaires • Semi-structured interviews During the intervention (6 weeks): • Participatory observation • Portfolios • Classroom artefacts Post-test: • Questionnaires • Semi-structured interviews Follow-up test: • Semi-structured interviews (6 months after the intervention)	1) A general introduction to nature of science 2) Reflection on the tentativeness and durability of scientific knowledge 3) Reflection on the tentativeness and durability of scientific knowledge 4) Non-contextualized activities 5) Application to chemistry teaching 6) Media reflection about the tentativeness and durability of scientific knowledge

## Study 1: Conceptions About Tentativeness, Their Origins and Their Resistance to Change

In 2018, 50 German pre-service chemistry teachers (24 female, 24 male, two did not specify; average age: 22.9 years) took part in an exploratory study as part of two parallel chemistry education courses. The primary aim of this study was to answer the first three research questions (cf. Section 3.3). To determine each participants’ conceptions about tentativeness of scientific knowledge, multiple instruments (questionnaires, interviews, portfolios and participatory observations) were used, which are described in the following section (see Section 4.1). It should be noted that the reflection about scientific knowledge and scientific inquiry is highly relevant in chemistry teacher education at the university where the study was conducted. Thus, 19 of the 50 participants stated that they already completed a course about NOS prior to the study.

### Research Design and Methodology of Study 1

First, the future chemistry teachers completed a semi-standardized open-ended questionnaire as a pre-test, based, in part, on the VNOS-C questionnaire (Lederman et al., 2002; questions 1, 4 and 9 from VNOS-C were included, with question 4 modified to also include other types of scientific knowledge besides theories). Additionally, semi-structured interviews were conducted with five participants for validation and to generate more profound insights. In the sense of data and methodological triangulation (Flick, 2018), classroom artefacts, portfolios and participatory observation were collected during the following intervention to validate pre-service teachers’ views. Finally, the participants completed the same questionnaire as post-test, to determine conceptual changes. Correspondingly, the 5-week intervention between pre- and post-test was designed in the sense of the conceptual change theory by Posner et al. (1982) and was therefore divided into three stages: In the first stage, during the first unit of the intervention, the participants were instructed to reflect on their own preconceptions. Therefore, the pre-service teachers investigated characteristics of science with the help of literature from epistemology and science education research (Allchin, 2013; Driver et al., 1996; Lederman, 1992; Lederman et al., 2002; Maeng & Bell, 2013; McComas, 1998; Neumann & Kremer, 2013; Osborne et al., 2003; Popper, 1959; Reiners, 2017). Throughout the second stage (units 2, 3 and 4), they should rearrange their views or generate new ideas about the tentative nature of various types of knowledge. For this purpose, they analysed and reflected historical case studies from chemistry, for instance the replacement of phlogiston theory, the development of atomic models or the discovery of the first noble gas compounds. These topics were selected to illustrate the tentativeness of chemical knowledge, because all of them are directly related to the German curriculum of the school subject chemistry. Thus, a domain-specific approach to teach the tentative nature of science (Niaz, 2016) was applied. Since it is more easy to recognize tentativeness in relation to historical cases, the participants also dealt with more contemporary case studies, such as the discovery of quasicrystals. Additionally, they carried out non-contextualized, “hands-on” black box activities, designed to illustrate different aspects of NOS (Lederman & Abd-El-Khalick, 1998). During the third stage of the intervention (unit 5), the participants had the task to apply their new or extended conceptions to chemistry teaching. Accordingly, the future chemistry teachers analysed classroom situations and textbooks in which the tentativeness of science plays a key role. Six to twelve months after the intervention, follow-up interviews were conducted with four participants to determine whether achieved conceptual changes are also sustainable in the long term.

To answer the research questions presented in Section 3.3, pre- and post-test data from each participant were analysed using the qualitative content analysis according to Mayring (2015). In total, *n*_1_ = 41 participants completed both the pre- and post-test questionnaires. Based on comparable studies (Desaulniers Miller, et al., 2010; Mesci & Schwartz, 2017), the pre-service teachers’ views about tentativeness were assigned to six different categories. These categories form a continuum from naïve and inconsistent to increasing levels of “informed” (+ , + + , + + +). A participant’s view about the tentativeness of scientific knowledge was encoded as “naïve”, when it is contrary to the aforementioned guiding framework of the tentative and durable nature of science (cf. Section 2.4), for example, if a pre-service chemistry teacher regards scientific knowledge or at least a category of scientific knowledge as unchangeable. Accordingly, a participant was assigned to this category, because of the following statement: “Not scientific laws. They cannot be revoked, because other theories are based on them.”(BK23Pre).^4^ If the participant’s conception partially corresponds to the guiding framework, but partially also contradicts it, it was classified to the category “inconsistent”. All participants, who agree with the guiding framework, were assigned as “informed”. The three levels of “informed” are distinguished by the respective way of argumentation of the participants to explain the tentativeness of scientific knowledge. This way, it should be possible to measure the extent to which pre-service teachers not only acquire declarative knowledge, but can also explain and justify their views. If the participant argues solely with the help of examples, she/he is classified as “informed ( +)”. If the explanation of tentativeness contains a single argument, it was coded as “informed (+ +)”. Finally, if a participant provides a multidimensional and differentiated explanation, she/he reaches the highest level “informed (+ + +)”. Lastly, if a pre-service teacher’s statement could not be assigned to any of the previously mentioned categories, it was classified as “not classifiable”. Definitions of each category as well as representative quotes of participants’ views about tentativeness are shown in Table 2.

**Table 2:** Definitions of categories and representative quotes of participants’ views about tentativeness

Category	Definition	Representative quote
Naïve	The pre-service teacher’s ideas contradict the guiding framework: Scientific knowledge or at least types of scientific knowledge are regarded as unchangeable	“Not scientific laws. They cannot be revoked, because other theories are based on them.” – BK23
Inconsistent	The pre-service teacher’s ideas partly correspond to the guiding framework, but partially also contradict it	“In science, it is possible to prove hypothesis. […] Yes, the atomic model has changed again and again.” – KH44
Informed +	The pre-service teacher principally agrees (without justification) that scientific knowledge is tentative and provides related examples where appropriate	“Yes, it can change! The idea about the structure of an atom has also changed again and again: Rutherford, Thomson, Bohr, atomic orbitals.” – CW87
Informed + +	The pre-service teacher explains the principle tentativeness of scientific knowledge of all types *either* by improving experimental methods/obtaining new knowledge *or* by reinterpreting existing evidence and knowledge	“Anytime, the rule of falsification applies. Nothing is proven, it is just not yet disproved. The most prominent example is the phlogiston theory, which was consensus for a long time but has been refuted.” – GB22
Informed + + +	The pre-service teacher explains the principle tentativeness of scientific knowledge of all types by improving experimental methods/obtaining new knowledge *and* by reinterpreting existing evidence and knowledge	“Yes, as a result of “new” findings, namely new measurement results or due to new perspectives of scientists.” – TK06
Not classifiable	The pre-service teacher’s ideas could not be assigned to any of the previously mentioned categories	“-” – SK54Pre

The inter-coder reliability concerning views about tentativeness between the first author and a second researcher is *p*_0_ = 0.90, while the coefficient *κ* (Brennan & Prediger, 1981) is 0.86.

### Results of Study 1

In the following sections, main results of the first qualitative empirical study will be summarized and discussed in order to answer the first three research questions. Results of participants’ response classifications for both the pre- and post-test are presented in Table 3. To compare the results from pre-test with those of the post-test, only those pre-service teachers were considered who participated in both tests (*n*_1_ = 41).

**Table 3:** Categorization of pre-service teachers’ views in relation to tentativeness of scientific knowledge in study 1 (*n*_1_ = 41) (see Müller & Reiners, 2020b)

	Not classifiable	Naïve	Inconsistent	Informed		
				+	** + +**	** + + +**
Pre-test	1	4	10	13	12	1
Post-test	0	5	5	21	10	0

#### Research Question 1: Partially Informed and Inconsistent Views About Tentativeness

The pre-test results show that many pre-service chemistry teachers share the idea that scientific knowledge is principally tentative even before the intervention (26 out of 41). Accordingly, these pre-service teachers were classified as “informed”. However, many of the participants explained their views in this regard only with the help of examples and without more general arguments. For example, a participant with the code DB37 said: “They [scientific findings] are always temporary. […] For example, atomic models have been modified, supplemented, and concretized over time.” (DB37Pre). Accordingly, these “informed participants” (13 out of 26) were coded on the first level “ +” of the category “informed”. The example of the development and changes of different atomic models provided by DB37 is also by far the most frequently mentioned example of tentativeness by the pre-service chemistry teachers (20 mentions in the pre-test). This might be an indicator that some topics contribute to a greater understanding of tentativeness than others do. However, it is also possible that this example is simply the one most familiar to the participants due to their experiences at school and university education (see Sections 4.2.2 and 4.2.3).

Some pre-service teachers (10 out of 41) also held inconsistent views related to the tentative nature of scientific knowledge. For instance, one pre-service teacher on the one hand said that scientific knowledge is tentative, because “the atomic model has changed again and again” (KH44Pre). On the other hand, he states, “In science, it is possible to prove hypothesis” (KH44Pre). Thereby, a context analysis of the participant’s portfolio and questionnaires shows that the term “prove” in the above statement is not used in a sense of everyday language, but is to be understood as a distinction to the humanities and thus in an absolute manner. In total, only four participants were coded as “naïve”, because they expressed the opinion that scientific knowledge is not tentative or at least that an unchangeable core of scientific knowledge exists. For example, one of them noted: “These theories can change. Laws, on the other hand, do not change” (GK59Pre).

Overall, German pre-service chemistry teachers seem to have similar views on tentativeness as German pre-service physics teachers (cf. Höttecke & Riess, 2007). In this regard, the sample size must be taken into account.

#### Research Question 2: Pre-service Teachers’ Views Were Shaped by Experiences from University, School and Media

In order to identify the origins of their views, the participants were also asked during the pre-test to share experiences that shaped their views about tentativeness or immutability of scientific knowledge. The inductively formed categories (Mayring, 2015) from their responses are shown in Table 4. They indicate that the origins of pre-service chemistry teachers’ views about tentativeness are relatively heterogeneous. However, the image of pre-service teachers, both those with informed and those with naïve views, is mainly shaped by university courses and lectures (27 out of 41 participants integrated this category into their response). In this regard, six pre-service teachers, which were coded as “informed” in the pre-test, explicitly described university courses about chemistry education as particularly formative. Additionally, different subjects and courses during school education also seem to play a major role in the development of conceptions about scientific knowledge (this main category was coded 18 times). Both findings indicate that the tentativeness of scientific knowledge should be discussed within science education at school as well as at university, to ensure that pre-service science teachers develop adequate ideas.

**Table 4:** Influences named by pre-service chemistry teachers (*n*_1_ = 41) shaping their views about tentativeness

Main categories (entries)	Subcategories (entries)
University (27)	Chemistry education courses (6)
	Chemistry courses (5)
	Studying Science (5)
	Biology courses (2)
	Statistics course (1)
	University in general (8)
School (18)	Chemistry lessons (5)
	Science lessons (3)
	Biology lessons (2)
	Lessons in physics (1)
	Philosophy lessons(1)
	Pedagogy lessons (1)
	School in general (5)
TV shows and movies (12)	TV shows for children (7)
	Documentaries (3)
	Fiction (1)
	TV shows and movies in general (1)
Literature (8)	Scientific literature (4)
	Textbooks (2)
	Books in general (2)
Persons (7)	Working groups at university (2)
	Lecturers (2)
	Chemistry teacher (1)
	Physics teacher (1)
	Discussions on websites (1)
No entry (7)	

In addition to experiences from their time at university and school, the pre-service chemistry teachers’ responses reveal that they also perceive movies and TV shows as well as printed media as sources of their conceptions about tentativeness (see Table 4). Thus, in addition to scientific literature, textbooks and documentaries, they also mentioned television shows initially developed for entertainment such as the American sitcom “The Big Bang Theory”. Furthermore, they mentioned TV shows, which deal with scientific questions and answer them for children, for example “Mouse TV” (original: “Die Sendung mit der Maus”). The movies, TV shows and books listed by the pre-service teachers as influences on their conceptions about tentativeness of scientific knowledge might be useful in further studies, to create cognitive conflicts and initiate conceptual changes according to tentativeness.

#### Research Question 3: Views About Tentativeness Are Resistant to Change

After the intervention, more pre-service chemistry teachers possess adequate views about tentativeness than in the pre-test (31 instead of 26, see Table 3). In addition, the examples mentioned by the participants to explain tentativeness indicate that they associate their ideas more often with chemical content. However, due to the Wilcoxon test (Wilcoxon, 1945), these conceptual changes are not significant. Thus, although participants generally describe that scientific knowledge is tentative, they are still not able to explain their views and therefore mainly remain on the first level of “informed”. For example, student DB37 explains the tentative nature of scientific knowledge in the post-test again exclusively by means of examples: “Scientific findings can change. Science is always tentative. For example, in the past, the smallest possible charge was assumed to be the elementary charge e^−^. However, later it was discovered that there are even smaller charges. […] Another example would be all the atomic models that are revised again and again. This also applies to bonding models or new elements in the periodic table […]” (DB37Post). While DB37 thus provides new examples to explain tentativeness compared to the pre-test (cf. Section 4.2.1), he still lacks profound reasons and general arguments to justify the principle tentativeness of all scientific knowledge. Accordingly, he was again coded as “informed +”.

In addition, the participants’ examples and explanations, which were summarized according to the rules of inductive categorization (Mayring, 2015; see Table 5), indicate that their conceptions about tentativeness are often restricted to models and theories. By far, the most frequently named example in this regard was the development of atomic models (this category was coded 20 times), which most participants were familiar with due to their experiences from chemistry classes in school. In the post-test, there is an improvement concerning other categories of changeable knowledge. For example, laws, constants or conceptions are mentioned more often as subject to change in the post-test (see Table 5). Nevertheless, theories and models remain the most frequently mentioned examples. Accordingly, participant DB37 states in the post-test: “Before this course I was not aware that even constants are still able to change” (DB37Post). These findings suggest that pre-service chemistry teachers consider laws and other types of scientific knowledge to be relatively more durable than theories and models.

**Table 5:** Categories of changeable scientific knowledge mentioned by pre-service chemistry teachers (cf. Müller & Reiners, 2020b)

Categories of scientific knowledge mentioned	Pre-test	Post-test
Theories/models	41	63
Laws	8	13
Definitions/concepts	4	27
Hypotheses	4	22
Classification systems	4	3
Data	4	1
Universal constants	0	14

Furthermore, the intensive discussion of tentativeness seems to unsettle some pre-service teachers. For example, one participant describes his inner conflict after the intervention as follows: “Before [the intervention], I was unsure about whether it is possible to reject established knowledge, but now I think: Everything could be wrong, everything could be right. No one knows.” (LP05Post). This unsettlement of the participants provides an explanation for the fact that the categories “informed + +” and “informed + + +” decreased between the post-test and the pre-test (see Table 3). Additionally, it indicates that the short intervention is suitable to initiate the cognitive conflict necessary for a conceptual change (Posner et al., 1982), but it is not sufficient to solve it. Therefore, the conducted intervention could be regarded as a first step, but not yet as a reliable path towards a conceptual change regarding views about the tentative nature of scientific knowledge. This is also supported by the finding that the participants who had already completed a course about NOS prior to the study (16 out of 41) were not significantly more informed about tentativeness in the pre-test than the participants without prior knowledge were. However, the pre-service teachers who had some prior knowledge of NOS increased their knowledge significantly during the intervention compared to the other participants.

Overall, the views about tentativeness from most of the participants (18 out of 41) were assigned to the same category in the post-test as in the pre-test. While only nine participants reached a category on a “higher informed” level, conceptions of 13 pre-service teachers related to tentativeness were even coded at a “less informed” level in the post-test. Therefore, the resistance to change of pre-service chemistry teachers’ conceptions about tentativeness of scientific knowledge could be confirmed (cf. Mesci & Schwartz, 2017). In summary, three different aspects of this resistance can be found: First, the future chemistry teachers are not able to explain their views. Second, their conceptions about tentativeness are often restricted to models and theories and less to other types of scientific knowledge. Last but not least, the relatively short intervention about tentativeness unsettles some participants.

Consequently, a longer and more differentiated discussion of tentativeness of all types of scientific knowledge is necessary to achieve a more adequate understanding. And to make it less likely that pre-service chemistry teachers will be unsettled by such a discussion of tentativeness, the focus should also be on the certainty and durability of scientific knowledge. This stresses Clough’s (2007, p.2) statement: “Students who claim that science is tentative without acknowledging the durability of well-supported science knowledge can hardly be said to understand the nature of science”. In a subsequent study, these considerations were taken into account.

## Study 2: Testing and Evaluating Learning Environments Concerning Tentativeness

In summer 2019, a second qualitative study was conducted with 56 German pre-service chemistry teachers (29 female, 26 male, one person did not specify; average age: 21.5 years) in two parallel chemistry education courses. This second study was conducted primarily to answer the fourth research question, whether school-related contexts and NOS activities are suited to promote a more adequate and more functional understanding of tentativeness among future chemistry teachers (cf. Section 3.3). Additionally, it should confirm the results from study 1 regarding the German pre-service chemistry teachers’ initial conceptions about the tentativeness. Therefore, the used methods for data collection and data analysis remained mostly the same as in the first study (see Section 3.3, Table 1), with the exception of one new item in the questionnaires (see Section 5.1). However, in order to answer the fourth research question, the intervention was redesigned: First, in addition to the tentative nature of science, the focus of the intervention was also on the durability and credibility of scientific knowledge. Second, the intervention was extended to six sessions. While the reflection on case studies of the tentativeness of scientific knowledge in study 1 only took up one unit of the intervention, the participants of study 2 reflected on the tentativeness but also on the durability of scientific knowledge for two units (cf. Section 3.3, Table 1). Third, to deal with the resistance to change of ideas about tentativeness, which was confirmed in the first study, and in addition to the greater number of case studies from chemistry (see Section 5.2.3), new approaches and innovative learning arrangements were tested: a new non-contextualized, “hands-on” activity called “BlackTube” (see Section 5.2.1), an aid for structuring the tentativeness and durability of scientific knowledge (see Section 5.2.2), and a media reflection (see Section 5.2.3). These new methods and activities as well as the methodological changes will be explained in the following sections.

### Research Design and Methodology of Study 2

The second study aimed at answering the fourth research question, whether school-related contexts and NOS activities are suited to promote a more adequate and more functional understanding of tentativeness (cf. Section 3.3). Therefore, participants’ ideas about tentativeness were collected again with the help of pre- and post-test questionnaires, interviews with five participants, classroom artefacts, portfolios and participatory observation and were later analysed using the same categorization system as in study 1 (cf. Section 4.1). Based on 12 coded questionnaires, the inter-coder reliability concerning views about tentativeness between the first author and a second researcher is *p*_0_ = 0.88, while the coefficient *κ* (Brennan & Prediger, 1981) is 0.84. According to Landis and Koch (1977), this result could be described as an “almost perfect” (p. 165) agreement. After a discussion of all controversial cases, in which both coders were able to agree on one coding judgement in each case, perfect agreement was achieved in another run of coding the questionnaires.

The questionnaires used in study 2 mainly contained the same items as in study 1. Only one item was added, in which the participants are asked to state along five-point Likert rating scales, whether they consider different types of scientific knowledge to be more tentative or more durable (see Section 5.2.4).

### Research Question 4: A Combination of Different Approaches Could Promote a More Adequate and More Functional Understanding of Tentativeness

Results of the classification of pre-service chemistry teachers’ views in relation to tentativeness for both the pre- and post-test of study 2 are presented in Table 6. Thereby, only those participants were considered who participated in both tests (*n*_2_ = 47).

**Table 6:** Categorization of pre-service teachers’ views in relation to tentativeness of scientific knowledge in study 2 (*n*_2_ = 47) (see Müller, 2021)

	Not classifiable	Naïve	Inconsistent	Informed		
				+	** + +**	** + + +**
Pre-test	0	5	12	17	12	1
Post-test	1	0	5	11	16	14

At the beginning of the study, a lot of pre-service chemistry teachers again hold partially informed or inconsistent views about tentativeness (see Table 6). Views of five participants were also coded as “naïve”. This is in line with the results of the pre-test from the first study (cf. Section 4.2.1). After the intervention, however, views are significantly more informed this time: no pre-service teacher still possesses naïve views about tentativeness during the post-test, whereas views of 14 participants were even categorized on the highest level “informed + + +”, because these participants were able to justify the tentative nature of scientific knowledge based on various arguments. In comparison, no participant reached this level in the post-test of the first study. Additionally, in contrast to the first study, the majority of participants (29 out of 47) increased their level of understanding during the course. The significance of changes in pre-service chemistry teachers’ conceptions between pre- and post-test was also confirmed for the entire sample with the Wilcoxon (1945) test for dependent samples.

Regarding these results and the fourth research question, it can be stated that the different contexts and NOS activities used during the intervention in study 2 are able to promote a more adequate understanding of tentativeness in general. However, the qualitative analysis of the participants’ statements from the questionnaires and interviews reveals that the participants attribute different aspects to their conceptual change or growth. On the one hand, they mostly point out that structuring the course content with the help of a mind map was very useful (38 out of 47). On the other hand, they name non-contextualized activities, which were used during the intervention (40 out of 47) as well as current and historical case studies from chemistry as helpful (33 out of 47). Since the treated non-contextualized activities are for the most part already tried and tested activities (Lederman & Abd-El-Khalick, 1998), the following descriptions are limited to the newly developed activity “BlackTube” (see Section 5.2.1), a structuring aid (see Section 5.2.2), and different case studies from chemistry discussed during the intervention (see Section 5.2.3). However, it should be noted that the following descriptions are intended to show the opportunities of the respective methods and instructions. These descriptions, which are based on exemplary statements of participants, can by no means prove an individual success of any of these methods. Rather, it must be assumed that the various methods are successful for conceptual growth altogether. In addition, it should be noted that the longer intervention time compared to the previous study could also be a factor for the increased conceptual growth.

#### The BlackTube Activity

**Fig. 2:**
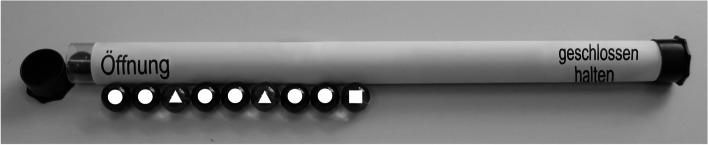
The BlackTube. A possible sequence of marbles within the tube is blue, blue, red, blue, blue, red, blue, blue, green. In the figure, different shapes represent the marble colours, as they would otherwise not be recognizable due to the black and white colouring: circles for blue, triangles for red and squares for green. On the tube, you can see the German instructions for “opening” (on the left) and “Keep closed” (on the right) (see Müller & Reiners, 2020a)

The BlackTube is an opaque tube, closed on both sides, in which different coloured marbles are lined up in a row (Müller & Reiners, 2020a). An example of such a construction is shown in Fig. 2. The task for students is to take the yet unknown objects out of the tube one by one and to describe each one. Based on these observations, they should make assumptions about what the next item inside the tube will look like. In this way, they can reproduce the aspect of finding patterns and regularities, which is central to science (ibid.).

The sequence of different coloured marbles inside the tube should be chosen in such a way that the assumed pattern has to be revised several times by the students during their investigation. In this way, learners can comprehend the continuous development of laws in chemistry. For example, in the intervention of study 2, the used sequence was “blue, blue, red, blue, blue red, blue, blue, green…”. Accordingly, after the first two marbles (which are both blue), most of the pre-service teachers assumed that only blue marbles were hidden inside the tube. In the further procedure, however, they predicted a different pattern of marbles, for example in the form of “blue, blue, red” or “blue, blue, different colour”. During the investigation of the BlackTube, it is crucial that at least the last marble always remains inside the tube. This should symbolize the principle tentativeness of scientific laws and regularities, because, in this way, an absolute prove of all cases in the sense of the principle of falsification (cf. Section 2.1) is not possible for the learners. Since students are otherwise free to end the investigation of the BlackTube whenever they want, different results can be obtained. In this way, the subjectivity of data interpretations as well as the procedure of finding a scientific consensus can be discussed.

While many black box activities focus on the properties of scientific models and theories (Lederman & Abd-El-Khalick, 1998), the BlackTube is especially developed to show the development and tentativeness of regularities and laws throughout the history of science. The activity thus complements the well-known black box activities (Müller & Reiners, 2020a). This is also confirmed by many participants of study 2. For example, one of the pre-service chemistry teachers notes in her portfolio that the BlackTube, in contrast to other activities, “impressively shows that some laws have to be discarded when new observations contradict the previous ones” (UD69). In addition, one participant states the BlackTube would shake up “the common view that laws are not refutable and permanent. This is done in a very structured, understandable, and also playful way” (SH26). Furthermore, the BlackTube is described as well suited to “address and clarify the durability of scientific knowledge” (RL76). Participant SD98, whose ideas were coded as “inconsistent” before the intervention and as “informed + + +” afterwards, names the “BlackTube” activity as the most helpful non-contextualized activity for his conceptual growth. In his portfolio, he describes the activity as follows: “With the help of a BlackTube, it is possible to illustrate the tentativeness of scientific laws and regularities. It also shows that a larger amount of data can lead to better results, but not to absolute certainty. If too few marbles are drawn out of the tube, the regularity cannot be captured correctly, because there is a lack of data. However, since not all marbles are drawn, there remains uncertainty as to whether the regularity […] is universal or if it is just ‘random’.” (SD98). Therefore, the BlackTube activity provides an opportunity for science educators to discuss the relationship between data and evidence, which leads to the durability of scientific knowledge (Cobern et al., 2022; cf. Section 2.3).

With regard to its use in chemistry school lessons, some pre-service teachers see an advantage of the BlackTube compared to other black box experiments for demonstration to young learners (Müller & Reiners, 2020a). On the other hand, one pre-service teacher warns about the possibility that learners could cheat during the BlackTube activity. For example, learners could take out all marbles and thus destroy the illusion that the sequence could not be determined absolutely. In total, 16 participants state in their portfolios that they would use the BlackTube activity in their future chemistry school lessons.

#### An Aid for Structuring the NOS Aspect of Tentativeness and Durability of Scientific Knowledge

**Fig. 3:**
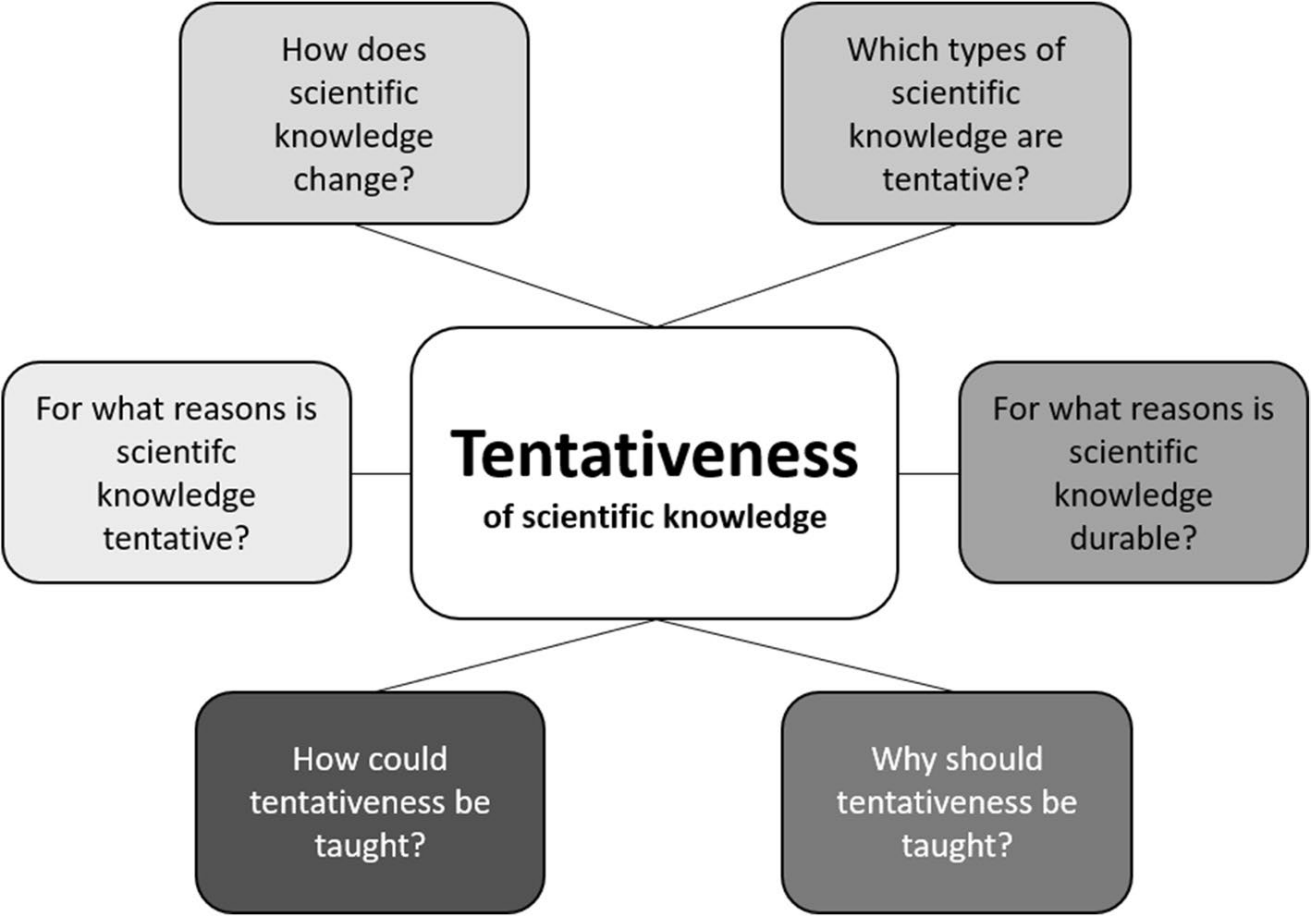
An aid for structuring the NOS aspect of tentativeness and durability of scientific knowledge

At the beginning of the intervention, the participants got a structuring aid in form of a mind map (see Fig. 3). In this mind map, they were asked the most important questions about the tentative nature of scientific knowledge from the perspective of philosophy of science as well as from the perspective of science education. For example, the structuring aid includes the questions, which types of scientific knowledge are tentative and for what reasons. On the other hand, they were asked how tentativeness could be taught to students. This should lead to discussions about instructional strategies and representations to teach the tentative nature of science (Mesci et al., 2020). In order to make it less likely that participants would be unsettled by such a discussion of tentativeness, they were also asked about reasons for the durability of scientific knowledge. At the beginning of the intervention, the participants were supposed to answer these questions. During the course, they were asked to gradually complete and revise the structuring aid. In this way, the participants were able to get an overview and network about the intervention and its content. On the other hand, the structural aid should make their own learning process transparent.

The following statement of a participant shows that the structuring aid can help pre-service chemistry teachers to recognize reasons for the tentative nature of science and to imprint them in their memory: “Before the course, I was already aware that science is mostly tentative, but now, with the help of the concept map, I understand how and why scientific knowledge changes” (SP11Post). Thus, with the help of the structuring aid, the participants from study 2 are able to justify the tentativeness in a more well-founded way. In addition, some statements indicate that the structural aid also leads to a greater awareness of reasons for the durability and credibility of science, such as empirical confirmation and peer review, and thus also prevents confusion on the side of the pre-service teachers: “I really liked the concept map […]. Especially, that it contained both: changeable and durable” (BN92). Thus, when teaching about tentativeness, care should be taken to address also the durability, reliability and credibility of scientific knowledge to the same extent. This confirms a hypothesis derived from results from the first study (cf. Section 4.2.3).

In addition, the structuring aid can also help to broad the perspective of some pre-service teachers in terms of different types of scientific knowledge, as the structuring aid of a participant exemplifies. When asked “Which scientific knowledge is tentative?”, the participant UA70 asks himself at the beginning of the intervention “All?” and initially seems to consider only hypotheses and scientific models as tentative. At the end of the intervention, he finally adds the note “All of them!” to his structuring aid.

#### Case Studies from Chemistry and a Reflection of Media

During the intervention of study 2, the participants dealt with numerous contemporary and historical case studies from chemistry, such as the demise of the phlogiston theory, the law of conversation of mass, the evolution of the periodic table of elements, an almost-forgotten acid–base definition by Mikhail Usanovich (Huheey, 1972; see Reiners et al., 2022) and the discovery of the first noble gas compounds as well as the discovery of quasicrystals. Most of the chosen examples have a connection to the German curriculum of the school subject chemistry. In addition, they were selected to show a wide range of reasons for changes of scientific knowledge. For example, universal constants could be specified with increasing precision due to technological progress, while the phlogiston theory was discarded because experimental findings were reinterpreted on the basis of new theoretical ideas. Furthermore, the case studies should demonstrate the credibility and workability of durable knowledge in chemistry. For example, in the course of time, all gaps in the periodic table were closed on the basis of its predictive power.

Additionally, the larger number of texts compared to the first study should illustrate the change (but also the robustness) of as many different types of scientific knowledge as possible. According to many participants, the different case studies are actually helpful to consider the principle tentativeness of all types of scientific knowledge. For example, participant SD98, who is able to justify tentativeness of scientific knowledge during his post-test, although he considered laws to be unchangeable in the pre-test, says “Above all, the examples and case studies helped me to rethink my point of view—by making clear what kind of knowledge could change.” (SD98Post).

The case studies dealt with should thus primarily serve as a tool for pre-service chemistry teachers to deal with the current socioscientific issues, challenges and public controversies. Afterwards, for example, the participants discussed how to deal with the current discussions on climate change in the context of chemistry lessons.

The follow-up interviews, which were conducted 6 months after the intervention, indicate that a media reflection arranged during the intervention also left a particularly lasting impression on the participants. Three out of the four interviewed pre-service teachers stated that they now watch movies or TV shows more critically and that they would almost automatically reflect, whether the respective movie or TV show conveys an adequate image of scientific knowledge or scientific inquiry. For example, SD98 states in the follow-up interview: “The media examples […] have led to the case that I can no longer watch any movie without having that in the back of my mind. […] Now I sometimes just sit there watching and shake my head. But it also shows how present science is in the world for everyone, and what kind of images about science is created.” (SD98follow-up).

#### Scientific Hypothesis, Theories and Models Are Regarded as “More Tentative” than Scientific Laws and Universal Constants

Before and after the intervention of study 2, participants were also asked to rate along a five-point Likert scale whether they considered various individual types of scientific knowledge to be more tentative or more durable. The results presented in Table 7 show that during the pre-test, pre-service chemistry teachers considered universal constants and scientific laws to be particularly durable. Conversely, scientific hypotheses as well as scientific theories and models were apparently considered to be especially tentative by most participants. The post-test results indicate that these tendencies are still present after the intervention. However, in the post-test, participants rated individual types of knowledge significantly less often as “absolutely durable”. The paired sample *t* test (Rasch et al., 2014) confirms significant differences (α = 0.05) between the participants’ assessments in the pre- and post-test according to scientific definitions and concepts, laws and universal constants as well as classification systems. The arithmetic mean of assessments for all these types of knowledge is smaller compared to the pre-test, so the pre-service teachers’ assessments for these items shifted more towards “tentative” on average. Remarkably, some participants assess scientific theories and models as “more durable” in the post-test. This can be interpreted as an indication that, after the intervention, the pre-service teachers have not only internalized the tentativeness, but also the durability of scientific theories and models. Accordingly, they were now increasingly of the opinion that an old theory is not simply discarded as soon as a new one is developed, but that the old theory can retain its explanatory power within an area of application: “I already said before that theories can definitely develop and change, but that they build upon each other and that old theories can still be correct and […] new ones have simply been added that explain some aspects better” (ID12).

**Table 7:** Assessments of pre-service chemistry teachers (*n*_2_ = 47) on the tentativeness and durability of different types of scientific knowledge during the pre-test and post-test of study 2 (see Müller, 2021)

	Pre-test					Post-test				
	Tentative				Durable	Tentative				Durable
Data	6	12	10	11	8	9	10	9	11	8
Definitions/concepts	6	8	13	18	2	6	17	13	9	2
Laws	0	5	4	17	21	2	4	12	27	2
Hypotheses	30	9	7	1	0	33	9	4	0	1
Universal constants	0	3	5	15	24	2	2	9	25	9
Classification systems	2	9	10	20	6	5	16	15	11	0
Theories/models	11	20	13	3	0	8	20	14	5	0

## Conclusions and Discussion

As shown in the literature review in this article, tentativeness is a decisive feature of scientific knowledge from the perspective of philosophy of science and science education (see Sections 2 and 3). Accordingly, an adequate understanding of this NOS aspect should be promoted within science education to enable students to actively participate in current debates about the credibility of scientific knowledge. However, the two presented empirical studies indicate that German pre-service chemistry teachers often possess inconsistent or only partially informed conceptions of the tentative nature of science, which are furthermore relatively resistant to change and often restricted to scientific theories and models. Additionally, pre-service teachers are often not able to justify their views about tentativeness and get unsettled by a longer discussion about it.

In order to promote a more adequate understanding within science education, new approaches were tested and evaluated during the second study, such as the non-contextualized NOS activity called “BlackTube” or a structural aid in form of a mind map. Furthermore, results of the conducted studies indicate that it is in general central to support pre-service teachers by structuring their conceptions about tentativeness. In this way, they can trace their own learning process. Additionally, such aids could help them to connect different reasons for tentativeness with each other. This seems to be important, because those reasons enable pre-service teachers and their future pupils to argue and participate in discussions about the scientific nature of statements in public discussions. To make it less likely that pre-service teachers will be unsettled by an intensive discussion of tentativeness, the durability and reliability of scientific knowledge should be addressed to the same extent. After all, pre-service teachers should not only educate their future pupils declaratively about the changeability of scientific knowledge, but rather promote the competence of these learners to be able to evaluate scientific findings and their credibility.

Due to the relatively small sample size and restriction to one location, the presented results cannot be generalized without further investigation. However, the second study emphasizes the importance of investigating students’ and pre-service teachers’ preconceptions. In this case, based on the identified conceptions, it became apparent that many German pre-service chemistry teachers already consider scientific theories and models to be tentative, because of their experiences from school science or university courses. The difficulty lays rather in recognizing and justifying the tentativeness of other types of scientific knowledge (cf. Table 7). Therefore, greater sensitivity according to different types of scientific knowledge is necessary in teaching tentativeness within chemistry teacher education. In this regard, it is also important to discuss the importance of such a differentiation of various types of scientific knowledge with pre-service teachers to increase their interest in teaching tentativeness and other aspects from nature of science. Accordingly, the question arises as to whether some of the common modelling approaches of NOS actually address the difficulties of pre-service science teachers in terms of tentativeness and durability of scientific knowledge or whether more detailed and differentiated theoretical approaches are still necessary. As a first step, a guiding framework of the tentativeness and durability of scientific knowledge for science teacher education was developed to support science teachers in designing adequate learning environments (cf. Section 2.4). Although the tested learning arrangements were initially developed particularly in relation to the tentative nature of science, the obtained results can probably also be transferred to other aspects of NOS, since tentativeness represents a particularly central feature of scientific knowledge.

## Footnotes

1 It should be noted that although the philosophy of chemistry is now an established field with its own history (Scerri & Fisher, 2016), instructions to teach epistemological aspects within science education are usually discussed in the context of nature of science (NOS), i.e. in relation to all natural sciences. Accordingly, selected positions from the philosophy of science and epistemology are presented in this section in order to illustrate the tentativeness of scientific knowledge.

2 In this regard, it is important to point out again that scientific theories do not have to be empirically tested in order to be acknowledged and accepted. For example, the string theory has an extremely important position in modern theoretical physics due to its explanatory potential without having been directly corroborated by any empirical findings (Dawid, 2006).

3 The nine analysed journals are Science Education, Science & Education, Journal of Research in Science Teaching, International Journal of Science Education, Research in Science Education, Journal of Science Teacher Education, International Journal of Science and Mathematics Education, School Science and Mathematics and Journal of Science Education and Technology (Cofré et al., 2019, p. 210).

4 The authors translated all German quotations in this article.
